# Genotype-Dependent Effects of COMT Inhibition on Cognitive Function in a Highly Specific, Novel Mouse Model of Altered COMT Activity

**DOI:** 10.1038/npp.2016.119

**Published:** 2016-08-10

**Authors:** Chris Barkus, Clio Korn, Katharina Stumpenhorst, Linda M Laatikainen, Dominic Ballard, Sheena Lee, Trevor Sharp, Paul J Harrison, David M Bannerman, Daniel R Weinberger, Jingshan Chen, Elizabeth M Tunbridge

**Affiliations:** 1 Department of Psychiatry, University of Oxford, Oxford, UK; 2 Medical School, University of Oxford, Oxford, UK; 3 Department of Physiology, Anatomy and Genetics, University of Oxford, Oxford, UK; 4 Department of Pharmacology, University of Oxford, Oxford, UK; 5 Oxford Health NHS Foundation Trust, Oxford, UK; 6 Department of Experimental Psychology, University of Oxford, Oxford, UK; 7 Lieber Institute for Brain Development, Johns Hopkins University, Baltimore, MD, USA; 8 National Institute of Mental Health, National Institutes of Health, Bethesda, MD, USA

## Abstract

Catechol-*O*-methyltransferase (COMT) modulates dopamine levels in the prefrontal cortex. The human gene contains a polymorphism (Val^158^Met) that alters enzyme activity and influences PFC function. It has also been linked with cognition and anxiety, but the findings are mixed. We therefore developed a novel mouse model of altered COMT activity. The human *Met* allele was introduced into the native mouse *COMT* gene to produce COMT-Met mice, which were compared with their wild-type littermates. The model proved highly specific: COMT-Met mice had reductions in COMT abundance and activity, compared with wild-type mice, explicitly in the absence of off-target changes in the expression of other genes. Despite robust alterations in dopamine metabolism, we found only subtle changes on certain cognitive tasks under baseline conditions (eg, increased spatial novelty preference in COMT-Met mice *vs* wild-type mice). However, genotype differences emerged after administration of the COMT inhibitor tolcapone: performance of wild-type mice, but not COMT-Met mice, was improved on the 5-choice serial reaction time task after tolcapone administration. There were no changes in anxiety-related behaviors in the tests that we used. Our findings are convergent with human studies of the Val^158^Met polymorphism, and suggest that COMT's effects are most prominent when the dopamine system is challenged. Finally, they demonstrate the importance of considering *COMT* genotype when examining the therapeutic potential of COMT inhibitors.

## INTRODUCTION

Catechol-*O*-methyltransferase (COMT) metabolizes dopamine: genetic and pharmacological reductions in COMT activity increase prefrontal dopamine transmission (Kaenmaki *et al*, 2010; Lapish *et al*, 2009; Tunbridge *et al*, 2004; Yavich *et al*, 2007). Thus, COMT inhibition is a potential therapeutic approach for the numerous psychiatric indications in which prefrontal dopamine is implicated (Robinson *et al*, 2012). Findings from humans (Apud *et al*, 2007; Farrell *et al*, 2012; Giakoumaki *et al*, 2008) and mouse models (Risbrough *et al*, 2014) suggest that the impact of COMT inhibition depends on functional variation within the *COMT* gene, consistent with the proposed inverted-U-shaped relationship between dopamine signaling and prefrontal-dependent task performance (Goldman-Rakic *et al*, 2000).

The valine-to-methionine (Val^158^Met) polymorphism in the human *COMT* gene directly affects the enzyme activity: Met homozygotes have ~40% lower COMT activity than Val homozygotes (Chen *et al*, 2004). Associations between Val^158^Met and a wide range of neuropsychiatric phenotypes have been investigated (Egan *et al*, 2001; Tunbridge *et al*, 2006, 2012). However, while some, notably associations between *COMT* Val^158^Met and dopamine-dependent cognitive function (Egan *et al*, 2001; Farrell *et al*, 2012) and anxiety-related phenotypes (Olsson *et al*, 2005; Pooley *et al*, 2007), show promise, non-replications exist; thus, associations between *COMT* and neuropsychiatric phenotypes remain controversial (Farrell *et al*, 2015). However, the functional genetic architecture of *COMT* has proved considerably more complex than initially appreciated (Gothelf *et al*, 2014; Nackley *et al*, 2006). Furthermore, the impact of the Val^158^Met polymorphism (and, by extension, other functional genetic variation in *COMT*) may also be modulated by environmental factors (Caspi *et al*, 2005; Ursini *et al*, 2011). Animal models are therefore essential to assess the effects of *COMT* Val^158^Met under controlled genetic and environmental conditions.

The human *Met* allele appears to be human specific (Lotta *et al*, 1995; Palmatier *et al*, 1999). Rodent COMT has activity similar to (Lotta *et al*, 1995; Risbrough *et al*, 2014) or higher than (Chen *et al*, 2004) that of the human ancestral Val isoform. Mice with genetically altered COMT activity have consistently shown alterations in at least some aspects of cognitive function. For example, *COMT* knockout mice show improvements, and *COMT*-overexpressing mice impairments, in tests of attentional set shifting and spatial working memory (Babovic *et al*, 2008; Papaleo *et al*, 2008; Simpson *et al*, 2014). Consistent with these earlier findings are data from recently developed, humanized COMT transgenic mice that carry the human Val- or Met-*COMT* open reading frames on a *COMT*-null background: humanized Met-COMT mice show superior spatial working memory, compared with Val-COMT mice (Risbrough *et al*, 2014). Furthermore, COMT inhibition improves cognitive function in wild-type rodents (Lapish *et al*, 2009; Tunbridge *et al*, 2004). COMT mouse studies also offer support for a link between low COMT activity and greater anxiety. The *COMT* knockout mouse shows increased anxiety and an exaggerated reactivity to acute stress, compared with wild-type animals (Desbonnet *et al*, 2012; Gogos *et al*, 1998; Papaleo *et al*, 2008, 2012). These findings are in keeping with the demonstration of reduced anxiety in one line of COMT-overexpressing mice (Papaleo *et al*, 2008), although a second, forebrain-specific *COMT*-overexpressing mouse line did not show this change (Simpson *et al*, 2014).

Here we describe a novel mouse model of altered COMT function, relevant to the human Val^158^Met polymorphism. We demonstrate specific influences on the function of COMT but crucially without notable effects on the expression of other genes (something which has not been studied in the other COMT mouse models thus far). We compared performance of COMT-Met mice with that of their wild-type littermates on cognitive and anxiety tasks that have previously been shown to be sensitive to the effects of COMT. We included the 5-choice serial reaction time task (5CSRTT), arguably the most widely used test of cortical function (Chudasama and Robbins, 2004), which has previously been associated with COMT activity (Grissom *et al*, 2015; Papaleo *et al*, 2012) (albeit not in all studies; Paterson *et al*, 2011). We also examined performance on hippocampal-dependent tests of spatial memory, given our previous demonstration that COMT inhibition increases spatial novelty preference, and may influence dopamine levels in this region (Laatikainen *et al*, 2012). Our results show that these mice have little cognitive or anxiety phenotype on the tests used, but that genotype differences emerge following pharmacological COMT inhibition.

## MATERIALS AND METHODS

**Figure 1:**
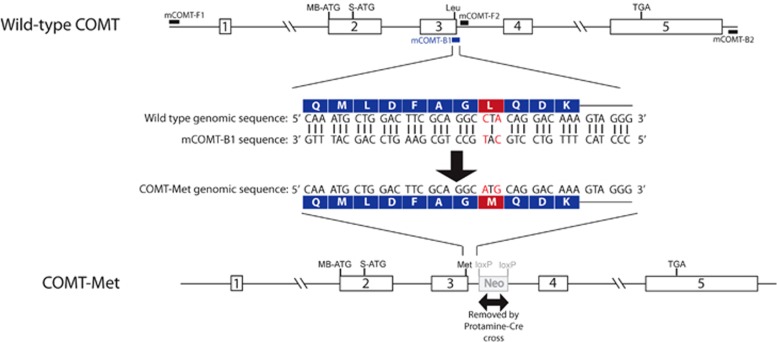
Generation of COMT-Met mice. The *Met* allele was knocked into the mouse *COMT* gene using a PCR-based strategy. The mouse COMT-B1 (mCOMT-B1) primer introduces the *Met* allele into the *mCOMT* gene (mismatched bases are highlighted in red). The final transgene contained the coding region of the *mCOMT* gene (amplified region: chr16:18 407 548–18 415 235, according to Mouse Genome December 2011 GRCm38/mm10 Assembly) with the *Met* allele, as well as a floxed PGK-neo selection cassette in the intron between exons 3 and 4. The selection cassette was subsequently removed by crossing the COMT-Met mice with a Cre recombinase-expressing line. COMT, catechol-*O*-methyltransferase. A full color version of this figure is available at the *Neuropsychopharmacology* journal online.

COMT-Met mice, in which the native COMT amino acid (Leu^148^) equivalent to the human Val^158^Met locus was replaced with a methionine, were generated using a PCR-based strategy at the National Institute of Mental Health, USA (see Supplementary Information and Figure 1), where all procedures were approved by the National Institute of Mental Health Animal Care and Use Committee and followed the *National Institutes of Health Using Animals in Intramural Research* guidelines. They were then shipped to the United Kingdom, where all procedures were carried out in accordance with the Animals (Scientific Procedures) Act 1986 and associated Home Office guidelines.

Details of immunoblotting, quantification of COMT enzyme activity, and neurochemical measures are included in the Supplementary Materials. Global gene expression was assayed in the frontal cortex, dorsal striatum, and nucleus accumbens using Affymetrix GeneChip Mouse 2.0 ST Array chips (Affymetrix UK, High Wycombe, UK), as described in detail in the Supplementary Information.

### Behavioral Testing

Full details of behavioral testing are provided in the Supplementary Information. Behavioral testing was conducted in COMT-Met mice and their wild-type littermates of both sexes from 9 weeks of age (*n*'s=10–20 per genotype group; mice backcrossed onto C57BL/6 background for 5–10 generations; Supplementary Table 4). Locomotor activity (total beam breaks) was recorded using the PAS Home Cage System (San Diego Instruments, San Diego, CA, USA) and divided into 5 min time bins for analysis. Mice completed tests of anxiety-related behaviors (the elevated plus maze, the open field, and the novelty-suppressed feeding task (hyponeophagia)) (Barkus et al, 2012) and memory (spontaneous alternation and spatial novelty Y maze, reference memory Y maze, object recognition, and the Morris water maze), as described previously (Barkus et al, 2012; Reisel et al, 2002; von Engelhardt *et al*, 2008). Attentional performance was assessed using the 5CSRTT, with mice completing a number of different stages (Bari *et al*, 2008). Drugs and their vehicles were administered in a fully counterbalanced manner (as was the saline injection *vs* non-injection control stages of the task). For these counterbalanced stages, data were expressed as a percentage of day performance on the day before the manipulation (see Supplementary Materials for full details). Experimenters were blind to genotype for all non-operant tasks.

### Data Analysis

With the exception of microarray data (see Supplementary Information) and neurochemical data (which were non-normally distributed and in which the effect of genotype was examined using Mann–Whitney *U*-tests), data were analyzed using analysis of variance (ANOVA) or the Student's *t*-test. For ANOVA, between-subjects factors of sex, genotype and cohort (where relevant), and within-subjects day/trial measures (where relevant, including trials nested within days in the case of the Morris water maze) were included. Huynh–Feldt correction was used where the data failed Mauchley's test of sphericity. The main outcome measures for each of the behavioral tasks are detailed in the Results section. With the exception of the microarray analyses, all analyses were conducted in SPSS Statistics version 20 (IBM, Portsmouth, UK).

## RESULTS

### Decreased COMT Enzyme Activity and Protein Abundance in COMT-Met *vs* Wild-Type Mice

As anticipated, COMT activity and protein abundance was reduced in COMT-Met mice, compared with wild-type mice. Abundance of both the soluble (S-COMT; Figure 1a) and membrane-bound (MB-COMT; Figure 1b) protein isoforms were reduced in COMT-Met mice in all brain regions examined, compared with their wild-type littermates (genotype main effects: F's>53; *P*'s<0.001), in the absence of other main or interactive effects (F's<2.0; *P*'s>0.16). Similarly, COMT enzyme activity was lower in COMT-Met homozygotes than in wild-type mice in all brain regions examined (genotype main effects: F's>4.1; *P*'s<0.049; Figure 1c), in the absence of other main or interactive effects (F's<2.0; *P*'s>0.1).

### The COMT-Met Transgene Does not have Off-Target Effects on Expression of Other Genes

It is important to rule out unintended, off-target effects of transgenic manipulations that might magnify or minimize observed genotype differences (Olson *et al*, 1996). This is particularly relevant in the case of COMT, as the chromosomal region in which it resides is hemizygously deleted in 22q11 Deletion Syndrome (22q11DS). 22q11DS is a neurodevelopmental condition associated with cognitive impairments and a significantly increased risk for developing schizophrenia (Karayiorgou *et al*, 2010). A number of the genes within the critical deletion region (of which COMT is one) are implicated in the pathogenesis of 22q11DS and the mouse equivalent region (16qA13) contains orthologs of most of them (Karayiorgou *et al*, 2010). Multiple genes contribute to the cognitive changes seen in 22q11DS model mice (Drew et al, 2011), and epistatic interactions are implicated (eg, interactive effects of COMT and proline dehydrogenase (PRODH) appear to underlie their spatial working memory deficits; Paterlini et al, 2005). We therefore investigated gene expression, using microarrays, in the nucleus accumbens, dorsal striatum, and frontal cortex of COMT-Met *vs* wild-type mice.

There were clear differences in gene expression profiles between brain regions (Supplementary Figure 2), but no genes showed differential expression between genotype groups in any region after correction for multiple comparisons.

We examined the expression of loci within the 22q11DS critical deletion region more closely using a very lenient, uncorrected threshold. Strikingly, only three significant changes (one per region, in three different loci) were found (Supplementary Table 1). For all three loci showing nominal significance, the direction of change differed across regions (ie, expression was increased in at least one region and decreased in at least one other region in COMT-Met *vs* wild-type mice), strongly indicating that these nominally significant differences reflect Type 1 errors (the number of nominally significant differences equates to 4.2% of the comparisons made and is therefore in line with the predicted rate of Type 1 errors for *α*=0.05). Thus, the COMT-Met transgene does not appear to have notable off-target effects on the expression of genes within the 22q11DS critical deletion region.

### Neurochemical Changes in COMT-Met Mice

**Figure 2:**
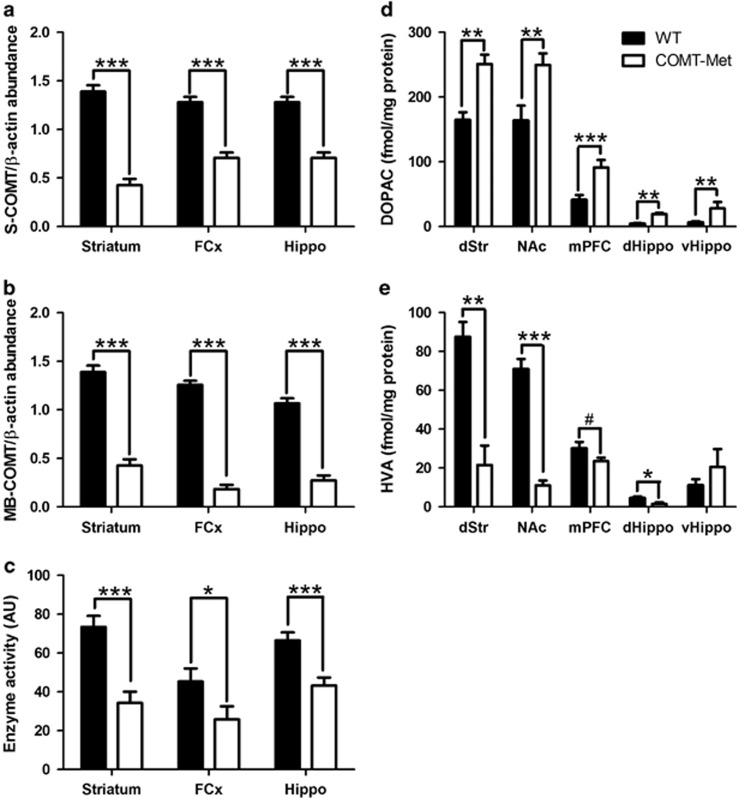
COMT-Met mice show robust reductions in COMT abundance and activity, and dopamine metabolism, compared with wild types. COMT abundance and activity is reduced in COMT-Met mice, compared with wild-type mice (WTs). (a) Soluble-COMT (S-COMT) abundance was reduced in COMT-Met mice (open bars) *vs* wild-type mice (closed bars) in the striatum (*n*=18 wild-type (9 male); *n*=19 COMT-Met (10 male)), frontal cortex (FCx; *n*=18 wild-type (8 male)); *n*=19 COMT-Met (10 male)), and hippocampus (Hippo; *n*=20 wild-type (10 male); *n*=20 COMT-Met (10 male)). (b) Membrane-bound-COMT (MB-COMT) abundance was reduced in COMT-Met *vs* wild-type mice in all brain regions (*n*'s as for S-COMT). (c) COMT activity was reduced in all brain regions in COMT-Met *vs* wild-type mice (*n*=20 wild-type (10 male); *n*=20 COMT-Met (10 male)). (d) 3,4-Dihydroxyphenylacetic acid (DOPAC) is increased in the dorsal striatum (dSt), nucleus accumbens (NAc), medial prefrontal cortex (mPFC), dorsal hippocampus (dHippo), and ventral hippocampus (vHippo) in COMT-Met mice (open bars) *vs* wild-type mice (closed bars). (e) Homovanillic acid (HVA) is decreased in all regions except the ventral hippocampus, in COMT-Met mice *vs* wild-type mice. *n*=8 wild-type (7 for vHippo); *n*=12 COMT-Met male mice. ***P<0.001; **P<0.01; *P<0.05; ^#^*P*<0.1. COMT, catechol-*O*-methyltransferase.

COMT converts 3,4-dihydroxyphenylacetic acid (DOPAC) to homovanillic acid (HVA), thus COMT-Met mice are predicted to show increases in DOPAC and reductions in HVA, indicating reduced dopamine metabolism, compared with wild-type mice. Consistent with this, COMT-Met mice showed increased tissue DOPAC levels in all regions examined (*P*'s<0.008; Figure 2a). Furthermore, compared with wild-type mice, COMT-Met mice also had reduced tissue levels of HVA (Figure 2b) in the nucleus accumbens, dorsal striatum, and dorsal hippocampus (*P*'s<0.017), and a trend level decrease in the frontal cortex (*P*=0.057). Tissue HVA levels were unchanged in the ventral hippocampus (*P*=0.90; Figure 2b). There were no between-group differences in tissue levels of dopamine (*P*'s>0.34), nor of 5-hydroxytryptamine (*P*'s>0.18) or its metabolite 5-hydroxyindoleacetic acid (*P*'s>0.18) (Supplementary Table 2). Thus, dopamine metabolism is selectively reduced in the COMT-Met mice compared with that in wild-type mice.

### COMT-Met Mice Show Normal Performance on Anxiety-Related Tasks

In contrast, no genotype differences were seen in anxiety-related behaviors examined. There were no main effects of genotype in the elevated plus maze (time spent in open arms: F_1,56_=0.97; *P*=0.33), the novelty-suppressed feeding task (F_1,56_=0.53; *P*=0.47), or the anxiogenic open field (latency to enter central region: F_1,56_=2.8; *P*=0.102; time spent in central region: F_1,56_=0.80; *P*=0.39), nor were there interactive effects involving genotype (F's<1.8, *P*'s>0.19) (including sex; see Supplementary Information and Supplementary Figure 3). Locomotor activity in a novel environment was also unchanged (see Supplementary Information).

### COMT-Met Mice Only Subtle Changes in Learning and Memory at Baseline

**Figure 3:**
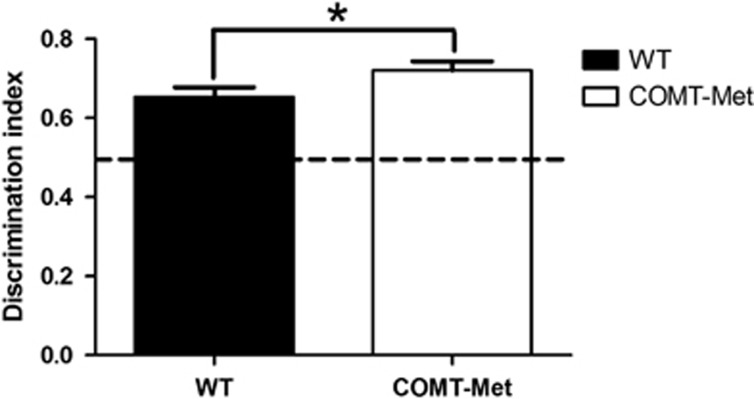
COMT-Met mice show greater spatial novelty preference than wild-type mice. The discrimination index was higher in COMT-Met mice (open bars; *n*=22 (13F/9M)) compared with their wild-type littermates (closed bars; *n*=17 (10F/7M)). The dotted line indicates chance performance. *P<0.05, one-tailed. COMT, catechol-*O*-methyltransferase.

We have previously demonstrated that spatial novelty preference is increased in rats given a COMT inhibitor, compared with vehicle (Laatikainen et al, 2012). Consistent with this finding, COMT-Met mice showed an increase in spatial novelty preference, compared with wild-type mice (*t*=−1.86; *P*=0.033 (one-tailed, based on *a priori* predictions from drug study; Laatikainen et al, 2012)) (Figure 3). However, there were no main effects of genotype on other tests of short-term memory (discrete-trial spontaneous alternation (F_1,35_=0.2, *P*=0.65); novel object recognition (F_1,35_=1.0, *P*=0.32)). Nor were there differences in associative, long-term spatial memory performance. There were no main effects of genotype on either the acquisition of the appetitively motivated reference memory Y maze task (F_1,38_=0.589, *P*=0.45; Supplementary Figure 4), or consistent changes on the aversively motivated Morris water maze task (F's<1.6; *P*'s>0.11; see Supplementary Information and Supplementary Figure 5).

### Genotype-Dependent Effects of Pharmacological COMT Inhibition on Performance of the 5CSRTT

#### Training and initial stages

Consistent with previous findings in COMT knockout mice (Papaleo *et al*, 2012), there were no differences between COMT-Met mice and their wild-type littermates on 5CSRTT performance during training (Supplementary Figure 6). The impact of different manipulations was then assessed (flanked on either side by a day run under standard conditions) as follows: Day 2, short stimulus duration (SD); Day 5, long intertrial interval (ITI); Days 8 and 11: saline injection *vs* no injection control (counterbalanced; to induce mild stress); Days 14 and 17, administration of tolcapone *vs* vehicle (counterbalanced; to study the response to pharmacological COMT inhibition); Days 20 and 23, administration of amphetamine *vs* vehicle (counterbalanced; to study the effect of dopamine release); Days 25–27, testing after free feeding. There were few effects of genotype on response to injection stress, amphetamine administration, and under free-feeding conditions (see Supplementary Information for details).

**Figure 4:**
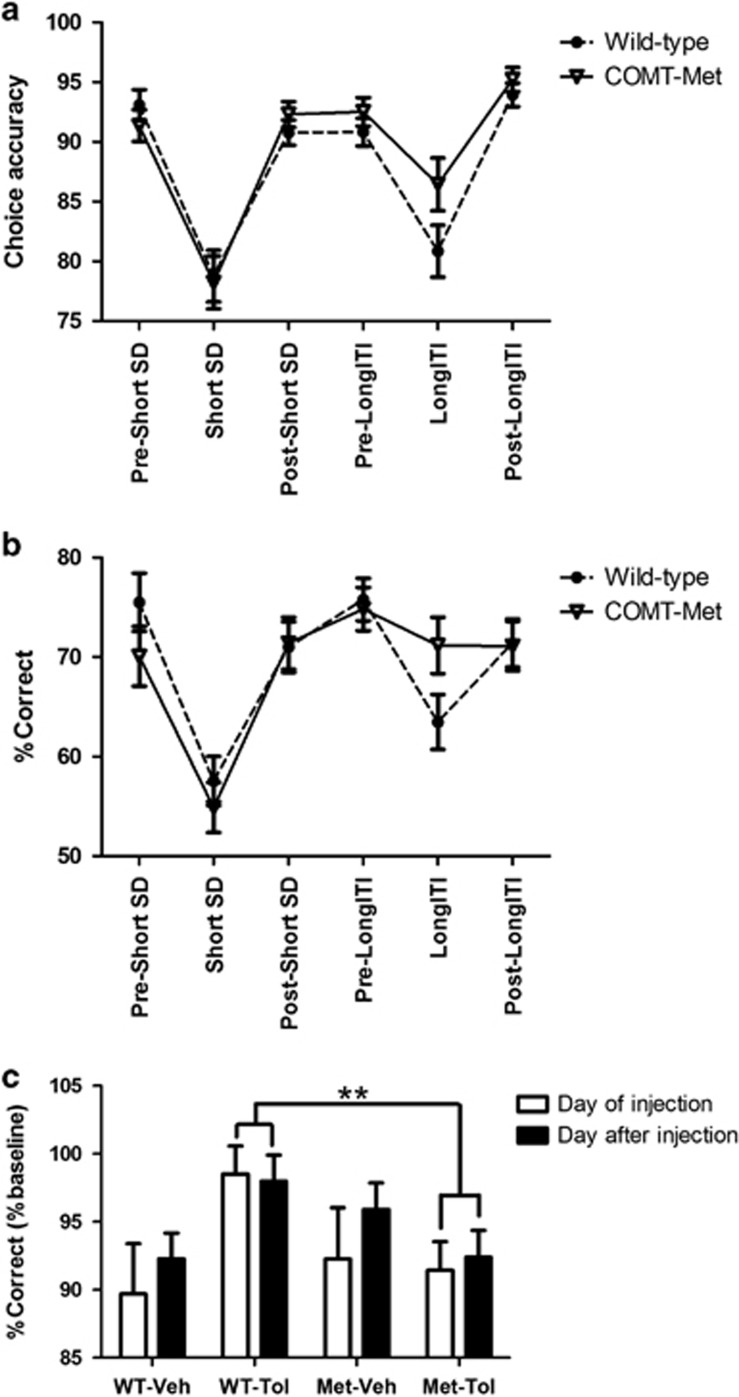
COMT-Met mice show changes in 5-choice serial reaction time task (5CSRTT) performance. Across the first 6 days, there were interactive effects of day and genotype on (a) choice accuracy and (b) %correct performance on the 5CSRTT that appeared to result primarily from trend-level differences in performance on the long-intertrial interval (ITI) stage, in which COMT-Met mice (open triangles, solid line) outperformed their wild-type littermates (closed circles, dashed line). (c) COMT-Met mice (Met) and their wild-type littermates (WT) showed differential effects of tolcapone (Tol) compared with vehicle (Veh) on 5CSRTT %correct performance. Specifically, following tolcapone administration wild-type mice outperformed their COMT-Met littermates. **P<0.01. Wild-type mice: *n*=20 (10F/10M); COMT-Met: *n*=20 (10F/10M). COMT, catechol-*O*-methyltransferase.

Performance across the first 6 days (encompassing a baseline day, and the short SD and long ITI manipulations) was examined within a single analysis, as for these stages all mice were exposed to the same manipulations in the same order (Table 1 and Figure 4). As well as main effects of day (choice accuracy: F_5,145_=62.1; *P*<0.001; percent correct (%correct): F_5,145_=31.2; *P*<0.001), there were genotype × day interactions for choice accuracy (F_5,145_=2.5; *P*=0.034) and %correct measures (F_5,145_=3.3; *P*=0.008), driven by trend-level genotype differences on the day of the long ITI manipulation (COMT-Met mice performed marginally better than wild-type mice: choice accuracy: *P*=0.081; %correct: *P*=0.061). There were no genotype differences on any other days (*P*'s>0.2). Genotype differences were not driven by motivational changes as there were no main or interactive effects of genotype on the latency to collect food rewards across these first 6 days of the test phase (F's<1.4; *P*'s>0.25). There were no other main or interactive effects (F's<1.4; *P*'s>0.23), other than a main effect of sex on %correct performance (females>males; F_1,29_=5.7; *P*=0.024).

### COMT-Met Mice Show Differential Effects of Pharmacological COMT Inhibition in the 5CSRTT

Consistent with human data, showing a beneficial effect of COMT inhibition by tolcapone in Val^158^ but not Met^158^ homozygotes (Farrell *et al*, 2012; Giakoumaki *et al*, 2008; Risbrough *et al*, 2014), tolcapone administration improved the performance of wild-type mice but not COMT-Met mice (Figure 4). Thus, there was a drug × genotype interaction for %correct performance (F_1,29_=6.2; *P*=0.019). *Post hoc* tests demonstrated that wild-type mice showed better %correct performance than COMT-Met mice following administration of tolcapone (*P*=0.005) but not vehicle (*P*=0.349). Accordingly, simple main effects analyses demonstrated that there was an effect of drug in wild type mice (performance after tolcapone better than after vehicle; F_1,29_=7.4; *P*=0.011) but not in COMT-Met mice (F_1,29_=0.64; *P*=0.43). These genotype differences were not driven by changes in motivation: there were no main or interactive effects involving genotype on the latency to collect the food rewards (F's<2.7; *P*'s>0.11), other than a trend-level day × genotype interaction (F_1,29_=3.9; *P*=0.059; detailed in the Supplementary Results), nor were there any other main or interactive effects involving genotype (including sex; see Supplementary Results for full statistical details). There were no main or interactive effects on the choice accuracy measure (F's>2.9; *P*'s>0.10).

## DISCUSSION

COMT-Met mice provide a novel and highly specific but subtle model of reduced COMT activity. COMT genotype had little impact on cognitive behaviors in the tests that we used; however, genotype differences emerge following COMT inhibition.

We introduced the human Met^158^ allele into the mouse COMT gene, which partially reduced COMT's abundance and activity and did not alter the expression of other genes. The COMT activity decrease resulted in reduced dopamine metabolism, indexed by an accumulation of DOPAC and a depletion of HVA, throughout the brain. The behavior of the COMT-Met mice was largely normal in the tests that we used, but genotype differences emerged after injection of the COMT inhibitor tolcapone. Specifically, following tolcapone administration, wild-type mice performed significantly better than COMT-Met mice on the 5CSRTT. Taken together, our results are highly consistent with data linking the human Val^158^Met polymorphism with cognitive function, in which findings of an association between Val^158^Met and baseline cognitive performance are mixed, whereas genotype-dependent effects of tolcapone are reliably observed.

### COMT-Met and Wild-Type Mice Provide a Highly Specific Model of Altered COMT Activity

The *COMT* gene is one of the most extensively studied in neuropsychiatry, in part, because the Val^158^Met polymorphism provides a proxy for enzyme activity. However, the relationship between Val^158^Met and behavior is complicated by the presence of other functional variants within the human *COMT* gene (Gothelf et al, 2014; Meyer-Lindenberg *et al*, 2006), and possibly also by gene–gene and gene–environment interactions (Caspi *et al*, 2005; Tan *et al*, 2007). Transgenic mouse models therefore provide an invaluable tool with which to investigate the effect of differences in COMT enzyme activity on a relatively homogeneous genetic background, and within a controlled environment.

A number of COMT mouse models have been developed. Two of them aim to model the effect of reduced COMT activity (which is of most direct relevance to the human Val^158^Met polymorphism, given that the ancestral form of COMT has high enzyme activity; (Chen *et al*, 2004)): *COMT* knockout mice contain a disrupted form of the *COMT* gene (Gogos *et al*, 1998), whereas humanized Val^158^Met mice carry the human Val- or Met-*COMT* open reading frames on a *COMT*-null background (Risbrough *et al*, 2014). Notably, both of these mouse models involve the deletion of the mouse *COMT* locus, raising the (as yet unexplored) potential for off-target effects on neighboring genes. Such effects might be particularly significant in the case of the COMT knockout mouse, as they would be anticipated to be present in knockouts but not wild-type mice. In the humanized mice, the native *COMT* locus is deleted in both Val- and Met-COMT mice, meaning that the presence of off-target effects should not directly confound genotype group comparisons. However, it is conceivable that the behavioral impact of differences in COMT activity might be magnified on such a background (eg, exaggerated effects of COMT inhibition have been reported in PRODH mutant mice; Paterlini *et al*, 2005). It will therefore be of significant interest to examine the expression of 22q11DS-equivalent genes in the existing COMT transgenic mice.

The genetic manipulation used to generate the novel mouse model described here represents only a small alteration to the native mouse genome, targeting a single amino acid within the mouse *COMT* gene. Consistent with this, the molecular phenotype of the COMT-Met transgene was highly specific: COMT abundance and activity and dopamine metabolism were robustly reduced in COMT-Met mice, compared with wild-type mice, in the absence of changes in the expression of other genes. Most critically, we show that COMT-Met mice have no notable alterations in the expression of *COMT*'s neighboring genes within the mouse equivalent of the human 22q11DS critical deletion region. Thus, we are confident that the behavioral and neurochemical phenotype of the COMT-Met mice results from specific changes in COMT activity. It should be noted, however, that despite our specific genetic alteration, the impact on COMT enzyme activity (<50% in the prefrontal cortex) is relatively modest, compared with some previous models (eg, knock outs), but perhaps more analogous to variation in enzyme activity associated with the Val^158^Met polymorphism in humans (Chen *et al*, 2004). Although the COMT-Met mice show robust reductions in enzyme activity, compared with wild-type mice, this results from a single base changes in the mouse *COMT* sequence, and so does not capture all of the polymorphic complexities of the human *COMT* gene (Nackley *et al*, 2006). Equally, however, it is unlikely that insertion of the human *COMT* sequence into the mouse genomic environment will fully recapitulate the human situation either. Ultimately, we argue that the most fruitful approach is to look for convergence between the various models. We believe that the enhancements of cognition associated with reductions in COMT function seen in the present model, although subtle, represent one such area of convergence.

### COMT-Met Mice Show Little Behavioral Phenotype at Baseline

The COMT-Met mice showed little, if any, behavioral phenotype on the tests that we used. Thus, the observed changes in baseline performance on the 5CSRTT (ie, on the long ITI stage) were marginal and, although COMT-Met mice outperformed their wild-type littermates on the spatial novelty preference task (consistent with our previous study showing that COMT inhibition increases spatial novelty preference; Laatikainen *et al*, 2012), their performance on other tests of short- and long-term spatial memory was unchanged. Our findings contrast with data from other COMT mouse models, which have shown consistent effects on cognitive function at baseline. However, these have been shown across a diverse range of tasks (including attentional set shifting and prepulse inhibition), several of which have yet to be studied in our model (Babovic *et al*, 2008; Papaleo *et al*, 2008,^2012^; Risbrough *et al*, 2014; Scheggia *et al*, 2014; Simpson *et al*, 2014).

It is conceivable that cohort differences in the number of times mice were backcrossed might mask some effects of the genetic manipulation in our model. However, we consider this unlikely for three reasons: first, epistatic effects within the 22q11DS-equivalent region (which could be present because of insufficient backcrossing) would be expected to magnify, rather than reduce, the effects of COMT (Paterlini *et al*, 2005); second, the few tests that were performed in mice backcrossed for five generations were replicated after backcrossing for a further three generations; third, the COMT-Met phenotype remained subtle even in the 5CSRTT performed in mice backcrossed for 10 generations onto C57BL/6 background (typically considered the ‘gold standard' Lusis *et al*, 2007) and in which we showed that there are no notable changes in gene expression.

The partial contrast between our findings and those observed in previous mouse models may relate, in part, to the magnitude of the effect of the transgene on COMT activity, as *COMT*-Met mice show an incomplete reduction in enzyme activity, compared with the total absence of COMT activity present in the *COMT* knockout in which the most striking phenotypes have been observed. Nevertheless, this is unlikely to be the entire explanation, as some cognitive changes have also been observed in heterozygous *COMT* knockout mice (Papaleo *et al*, 2012), and in humanized *COMT* mice, which show a more modest genotype difference in COMT activity (Risbrough *et al*, 2014). However, direct comparisons between the different mouse models are complicated by the inverted-U-shaped relationship between cortical dopamine signaling and cognitive performance: the magnitude of the *COMT* genotype effect is predicted to depend on the many factors that influence dopamine signaling, and, consequently, baseline levels of performance. For example, in the study of humanized *COMT* mice, levels of spontaneous alternation (which was used as the study's primary readout of working memory function) were markedly lower comapred with those observed here: both wild-type and COMT-Met mice in the current study showed ~90% alternation, whereas humanized Met-COMT mice showed ~70–80% alternation and humanized Val-COMT mice performed at chance levels (~50% alternation). Thus, it is unlikely that in the current study the COMT reduction present in COMT-Met mice could increase the already high levels of alternation seen in wild-type mice. It is notable in this regard that COMT-Met *vs* wild-type mice did show improved spatial novelty preference, a task that may tap into similar memory processes to spontaneous alternation, suggesting that the absence of a genotype effect on spontaneous alternation performance may indeed be due to ceiling effects. Finally, to our knowledge and as highlighted above, global gene expression has not been surveyed in any of the other COMT transgenic models; therefore, it is possible that the impact of lowered COMT activity is exacerbated in some of these models by effects on neighboring genes (Paterlini *et al*, 2005). It will therefore be of significant interest to assess gene expression, particularly in the 22q11DS-equivalent region, in other COMT mouse models.

### Genotype-Dependent Effects of COMT Inhibition on 5CSRTT Performance

The most striking aspect of our 5CSRTT findings is the differential effect of tolcapone on 5CSRTT performance in COMT-Met *vs* wild-type mice. Specifically, tolcapone improved performance in wild-type mice but not COMT-Met mice. This finding is consistent with data from human studies, in which Val^158^Met has been consistently shown to interact with the effects of tolcapone on working memory performance (Farrell *et al*, 2012; Giakoumaki *et al*, 2008), as well as with tolcapone-induced improvements in spontaneous alternation performance in Val- but not Met-COMT humanized transgenic mice (Risbrough *et al*, 2014). Thus, tolcapone improves working memory performance in high COMT activity individuals (eg, Val/Val humans; wild-type mice) (Farrell *et al*, 2012; Giakoumaki *et al*, 2008), while having little effect on (Giakoumaki *et al*, 2008), or even impairing (Farrell *et al*, 2012), performance in those with low enzyme activity. The presence of *COMT* genotype-dependent effects of COMT inhibition is consistent with the reported inverted-U-shaped relationship between prefrontal dopamine levels and cognitive performance (Goldman-Rakic *et al*, 2000). It is notable that we did not observe a similar drug by genotype interaction following amphetamine, nor was such an interaction observed in the *COMT* knockout mice (although this earlier study used lower doses of amphetamine and lacked a vehicle control, making direct comparisons problematic) (Papaleo *et al*, 2012). Similarly, human studies examining the Val^158^Met-dependent impact of amphetamine have showed much clearer interactive effects of genotype and drug on brain activation (determined by functional magnetic resonance imaging) than behavioral performance (Mattay *et al*, 2003). Although speculative, genotype-dependent behavioral effects might be more robust for tolcapone than amphetamine because of the relative specificity of tolcapone for cortical *vs* striatal dopamine transmission (Huotari *et al*, 1999; Tunbridge *et al*, 2004), compared with amphetamine (Hertel *et al*, 1995).

The consistency between our findings and those for the human Val^158^Met polymorphism, coupled with the subtlety and specificity of the genetic manipulation, means that the COMT-Met mice have significant potential utility for translational studies. Thus, as well as being useful for determining the neurobiological mechanisms underlying observed links between COMT and behavioral (and neuroimaging) measures in humans, they also have significant potential as a model system in which to investigate the possibility of *COMT* genotype-dependent effects of pharmacological compounds.

A notable aspect of our findings is the lack of evidence for sexually dimorphic effects of COMT in this model. A number of lines of evidence suggest sexual dimorphisms in some aspects of COMT's function (albeit these are not always statistically robust) (Tunbridge and Harrison, 2011). However, while we did observe sex differences in performance on a number of tasks (as detailed in the Supplementary Results), we did not find any reliable interactions between sex and genotype in our behavioral studies. This is consistent with the lack of any sex differences in COMT's abundance and activity in our model, as COMT's sexual dimorphisms are usually ascribed to estrogenic regulation. Thus, the COMT-Met mice do not appear to show sexual dimorphisms at baseline, although it will be of interest to see whether any emerge for other tasks, or under specific environmental conditions.

### No Gross Changes in Anxiety-Like Behaviors in COMT-Met Mice

We observed no increase in anxiety-related behaviors in the COMT-Met mice. Indeed, even where there were numerical differences between groups (none of which were close to statistical significance), COMT-Met mice demonstrated numerically *lower* levels of anxiety-like behaviors compared with wild-type mice. Based on the previous studies in COMT transgenic mice, an increase would have been anticipated (Desbonnet *et al*, 2012; Gogos *et al*, 1998; Papaleo *et al*, 2008). However, some of the earlier findings are sex specific (Desbonnet *et al*, 2012; Gogos *et al*, 1998) and not all COMT transgenic mice showed anxiety changes (Simpson *et al*, 2014). Similar complexities are seen in the human literature: while some studies linked the *Met* allele with anxiety disorders in humans (Woo *et al*, 2002) (albeit sometimes in men (Pooley *et al*, 2007) or women (Enoch *et al*, 2003) only), others found no association (Niarchou *et al*, 2014), and a recent meta-analysis of studies linking *COMT* with anxiety-related traits indicated a male-specific association with the Val (ie, the *high activity*) allele (Lee and Prescott, 2014). One possible reason for these apparently conflicting results is emerging evidence that associations between COMT and anxiety phenotypes may be complicated by the presence of gene–gene (Konishi *et al*, 2014) and gene–environment (Baumann *et al*, 2013) interactions. Data from COMT transgenic mice, in which genetic and environmental variability can be controlled and experimentally manipulated, are therefore invaluable for clarifying relationships between COMT and anxiety. The data presented here suggest that modest alterations in COMT activity do not alter gross changes in anxiety-like behaviors, at least in the tests used here. However, they do not preclude the possibility that COMT may influence anxiety phenotypes under specific genetic or environmental conditions.

## CONCLUSIONS

We demonstrate that, in a highly specific mouse model of altered COMT activity, *COMT* genotype has little impact on memory and attentional performance at baseline, but that genotype differences emerge following administration of the brain-penetrant COMT inhibitor tolcapone. These findings are consistent with human studies of the Val^158^Met polymorphism, in which data suggest that at baseline *COMT* genotype differences are only seen for relatively difficult working memory tasks (with high maintenance and manipulation demands), but that genotype differences emerge after tolcapone administration. Taken together, these findings demonstrate that the impact of *COMT* Val^158^Met may be more prominent for relatively challenging cognitive tasks, but that broader differences may emerge when the mesocortical dopamine system is challenged in some way. Here, we demonstrate that genotype differences emerge after tolcapone administration, but other genetic or environmental factors that also impact on prefrontal dopamine function would be expected to interact with *COMT* genotype differences in similar, nonlinear ways. Finally, our data provide further evidence for the importance of considering *COMT* genotype when investigating the therapeutic potential of COMT inhibitors.

## FUNDING AND DISCLOSURE

The authors declare no conflict of interest. This research was funded by the Intramural Research Program of the NIMH, Bethesda MD, USA to the Weinberger Laboratory, and by a UK Medical Research Council grant (G0700983) to PJH and EMT. Additional funding from Wellcome Trust studentships awarded to CK and KS, and a grant to EMT from the Royal Society (RG100516) and a Wellcome Trust Senior Fellowship (087736) awarded to DB. EMT is funded by a University Research Fellowship awarded by the Royal Society.

## Figures and Tables

**Table 1:** Summary of Behavioral Findings

**Test**	**COMT-Met phenotype**
Locomotor activity	No difference
Open field	No difference
Elevated plus maze	No difference
Novelty-suppressed feeding (hyponeophagia)	No difference
Spatial novelty Y maze	Greater novelty preference in COMT-Met mice than in wild-type mice
Reference memory Y maze	No difference
Spontaneous alternation	No difference
Novel object recognition	No difference
Morris water maze	No difference
5-Choice serial reaction time task	COMT-Met mice outperform wild-type mice at trend level on the long intertrial interval stage Wild-type mice but not COMT-Met mice performance improved by tolcapone administration

Abbreviation: COMT, catechol-*O*-methyltransferase.

## Supplementary Material

Supplementary Informations
