# Supplementary material for: Genotype-Dependent Effects of COMT Inhibition on Cognitive Function in a Highly Specific, Novel Mouse Model of Altered COMT Activity
Source: Neuropsychopharmacology. 2016 Aug 10;41(13):3060–9. doi: 10.1038/npp.2016.119 (PMC5101554; doi:10.1038/npp.2016.119)
Supplement: Supplementary Informations [file npp2016119x1.doc]

**Supplementary Methods**

Mice were bred in individually ventilated cages but were transferred to open cages after genotyping at approximately 7 weeks of age.

*Generation and development of COMT-Met mice*

A fragment of the COMT gene was amplified from mouse genomic DNA using Platinum Pfx high fidelity polymerase (Invitrogen, Life Technologies, Paisley, UK) and primers which introduced the Met allele into the position of the mouse COMT gene homologous to the human membrane-bound COMT (MB-COMT) codon 158 (Figure 1). The left arm of homologous mouse genomic DNA was cloned with the primers, mCOMT F1: 5’ CTC TTA AGT GCT AGA ATC TCA GA 3’ and mCOMT B1(Met): 5’ CCC TAC TTT GTC CTG CAT GCC TGC GAA GTC CAG CAT TTG 3’, which contains the Met mutation. The right arm of homologous mouse genomic DNA was cloned with the primers, mCOMT F2: 5’ CCA GTG TGG CAG CTG AGG GGT GAA GA A 3’ and mCOMT B2: 5’ CTC CCT TTT TGT TCT TGG GTG CAG A 3’. The left and right arm genomic DNA fragments were cloned into the pLoxP vector, which has two loxP sites flanking the PGK-neo selection cassette (a gift from Dr. Liya Sheng, NCI, NIH). The final construct, pCOMT-Met-LoxP-neo, was mapped by restriction enzyme digestion and confirmed by DNA sequencing.

Mouse embryonic stem (ES) cells were transfected with pCOMT-Met-LoxP-neo linearised with Pvu I. G418-resistant ES cell clones were screened by long-range PCR for homologous recombination at the COMT locus, using primers: COMT-ki-F1: 5’ GGT GCT CTT ACC CAC TGA GCC ATC TCA and neo-B1: 5’ GCT GCT AAA GCG CAT GCT CCA GAC T 3’. Positive ES cell clones were used for blastocyst injection (background strain: 129/sv) conducted by the NIMH Transgenic Core Facility. The microinjected blastocysts were implanted into pseudopregnant recipients. The genotypes of founder mice were confirmed by long-range PCR. Mice were backcrossed onto a C57BL/6J background for 3 generations. Homozygous mice were then crossed with mice expressing Cre recombinase under the control of the protamine promoter (129S/Sv-Tg(Prm-cre)58Og/J; JAX® Labs Strain: 003328, Bar Harbor, Maine, US) before backcrossing for a further 5-10 generations onto a C57BL/6J background. Mice were routinely genotyped using a restriction fragment length polymorphism assay: a fragment of the COMT gene was amplified using the primers Met-Mouse-Geno-F (5’ GCC AAA TCA TGG ATG CAG TGA TTC 3’) and Met-Mouse-Geno-R (5’ CTT TGG GGC TTT GGA GAG GAA 3’). The resulting 471bp amplicon was digested with StuI and the products visualized on 3% w/v agarose gels. The presence of the Met allele abolishes a StuI site, which cleaves the amplicon into 244bp and 227bp fragments in wild-type mice; thus, Met homozygotes show only a 471bp band, wild-type homozygotes show only 244bp and 227bp bands, and heterozygotes show bands at all three sizes.

*Quantification of COMT protein and enzyme activity in COMT-Met mice*

COMT-Met mice and their wild-type littermates were sacrificed (n=10 of each sex per genotype group). Frontal cortex, hippocampus and striatum were rapidly dissected on ice from COMT-Met homozygotes and their wild-type littermates and were snap-frozen on ice. Tissue was homogenized in 0.6% Tris and 50% glycerol buffer, containing protease inhibitors (cOmplete Protease Inhibitor Tablets, Roche, Mannheim, Germany). Protein concentrations were determined using the Bradford method and crude homogenates were stored in single use aliquots (at 1mg/ml concentration) at -80^o^C. COMT enzyme activity was determined in 20μg protein as described previously , except that incorporated tritium was determined in one-tenth of the total reaction volume, to minimize radioactive scintillation waste (values represent the mean of three replicates). COMT protein abundance was determined by immunoblotting in 4μg of crude protein homogenate (determined in pilot studies to be within the linear range for protein quantification for both COMT and β-actin). For some samples, insufficient protein remained for immunoblotting (see figure legend for n’s). Protein was separated on 4-20% Tris-glycine gels (Novex®, Invitrogen, Paisley, UK) and transferred to PVDF membrane (Immun-Blot®, Biorad, Hemel Hempstead, UK). Membranes were blocked in 5% w/v milk powder in phosphate buffered saline containing 0.2% Tween-20. COMT was detected using 1:10,000 dilution of anti-COMT monoclonal primary antibody (BD Biosciences Transduction Laboratories, Oxford, UK), followed by anti-mouse horseradish peroxidase secondary (1:5000; Biorad), and visualised using ECL Plus reagent (GE Healthcare, Little Chalfont, UK). Membranes were then stripped (Restore Stripping Buffer, Thermo Scientific, Hemel Hempstead, UK) and then re-probed to detect β-actin (as for COMT, but using 1:1,000,000 monoclonal anti-actin; Sigma Aldrich, Gillingham, UK). Films were digitized and densitometry was performed using ImageJ v1.47 (National Institute of Health, Bethesda, MD, US). MB-COMT (~28kDa) and S-COMT (~24kDa) bands were quantified separately, and were expressed as a proportion of the respective β-actin band.

*Neurochemical measures*

COMT-Met (n=12) and wild-type (n=8) mice (all male) were sacrificed and their brains were rapidly removed and snap-frozen in isopentane. Tissue punches were collected from frontal cortex, dorsolateral striatum, nucleus accumbens and dorsal and ventral hippocampus. Ventral hippocampal tissue was lost from one wild-type mouse. Tissue levels of dopamine, DOPAC, HVA, 5HT and 5HIAA were determined by high-performance liquid chromatography as described previously. The dopamine peak was masked in one of the ventral hippocampal samples from the wild-type group, so this sample was excluded. The amount of protein present in each tissue punch sample was determined using the Bradford method and results are expressed as fmol/mg protein. For 10 of the 12 COMT-Met mice (but none of the wild-types), dorsal hippocampal HVA was undetectable, these values were encoded as zero.

*Microarrays*

COMT-Met mice and their wild-type littermates were sacrificed and their brains were rapidly removed and snap-frozen in isopentane. Tissue punches were taken from frontal cortex, dorsolateral striatum and nucleus accumbens (shell) of 6 COMT-Met and 6 wild-type male mice. RNA was extracted using the Qiagen RNeasy Micro Kit (Qiagen Ltd, Manchester, UK) according to the manufacturer’s instructions. All samples were processed at the same time. RNA quality was assessed using Agilent 2100 Bioanalyser Nano-Chips (Agilent Technologies Inc, CA, USA). Only replicates with an RNA Integrity Number (RIN) of ≥6 were used, giving final numbers of 5 COMT-Met mice (6 for nucleus accumbens) and 5 wild-types. 1.5ng of RNA was reverse transcribed and amplified using the NuGen Ovation Pico WTA System V2 (NuGen Technologies Inc, CA, USA). Briefly, amplification is started at sites throughout the transcript, as well as at the 3’ end, thus reducing 3’ bias. The RNA was reverse transcribed to synthesise a cDNA with a SPIA® RNA tag sequence at the 5’ end of the cDNA. The cDNA is then amplified using the SPIA® process. Amplified, double-stranded cDNA was transformed into single strand sense. Fragmentation and biotinylation was subsequently performed using the Encore Biotin Module V2 (NuGen Technologies Inc). The sense cDNA was chemically and enzymatically fragmented to produce strands of 50-100bp in length. The fragmented sample was run on an Agilent Bioanalyser NanoChip (Agilent Technologies Inc) to confirm successful fragmentation. The strands were then labelled with biotin. Fragmented, labelled, single-strand sense cDNA was hybridised overnight at 45°C to the Affymetrix GeneChip Mouse 2.0 ST Array chip (Affymetrix UK Ltd, High Wycombe, UK). The chips were washed and then stained with streptavidin-phycoerythrin using the GeneChip Hybridization, Wash and Stain Kit (Affymetrix UK Ltd). The hybridised chips were scanned on the Affymetrix Gene Chip Scanner and signals computed using the GeneChip® Command Console® Software (Affymetrix UK Ltd). Quality control by the Command Console^TM^ 4.1.2 (Affymetrix UK Ltd) indicated that all samples were of sufficient quality for inclusion in the subsequent analysis. Data processing was performed using GeneSpring GX 12.6.1. The CEL files for individual brain regions were normalised in GeneSpring using Probe Logarithmic Intensity Error (PLIER). The gene expression values were then compared between COMT-Met and wild-type mice for each region using Linear Models for Microarray Data (LIMMA). Genotype group differences were also assessed using a moderated t-test with a Benjamini-Hochberg correction for multiple testing. Expression differences of genes falling within the 22q11DS critical deletion region were examined at a highly-lenient uncorrected threshold, to check for any suggestion of genotype differences.

*5-Choice Serial Reaction Time Task*

The 5CSRTT was performed in operant boxes (ENV-307W, Med Associates, St. Albans, VT, US). 5CSRTT training was performed as described previously . With the exception of the free-feeding stage, described below, mice were maintained on restricted food to maintain them at 85% of their free feeding weight. Following training, mice were tested for a further three days. Several manipulation stages were then performed. The final training settings (stimulus duration 0.8 sec, limited hold 7 sec, inter-trial interval 5 sec) were then used for all subsequent stages, except where otherwise stated. Stages were as follows:

1. Short stimulus duration (SD) stage (Days 1-3): Following a standard test day, the stimulus duration was reduced to 0.4 sec. All other settings remained unchanged. Stimulus duration was then returned to 0.8 sec for the session on the following day, in order to ensure any alterations were extinguished by returning to a longer stimulus duration.

2. Long Inter-trial interval (ITI) stage (Days 4-6): Following a standard test day, the ITI was increased from 5 to 7 sec. All other settings remained unchanged. The ITI was then returned to 5 sec for the session on the following day

3. Injection stress stage (Days 7-12): The effect of injection stress was assessed using a similar design to the Short SD and Long ITI stages, but conducted over six days, using the standard testing conditions. On the second and fifth days of the stage, half the animals received an injection (0.9% w/v saline, 1ml/kg i.p.) immediately before testing, while the other half did not. Injections were given in a Latin square design with the groups counterbalanced across genotype, sex and performance on day one. One (male COMT-Met) mouse failed to make any responses following saline injection and so was omitted from this stage of the analysis.

4. Tolcapone stage (Days 13-18): The same design (tolcapone and vehicle injections on the second and fifth days of the stage, administered in a fully-counterbalanced, Latin-square design) used for the injection stress stage was used subsequently to compare the effect of 30mg/kg tolcapone (i.p.; 5ml/kg) or vehicle (20% w/v cyclodextrin in 0.9% w/v saline), injected one hour prior to testing. Since tolcapone is a tight-binding inhibitor there is a very narrow range of drug concentrations over which there is a linear relationship between dose and COMT inhibition . We therefore used a single dose of tolcapone, which we (Stumpenhorst and Tunbridge, unpublished observations) and others have shown to robustly inhibit COMT activity in mice.

5. Amphetamine stage (Days 19-24): Mice were administered 1.0 mg/kg amphetamine (i.p.; 1ml/kg) or vehicle (0.9% w/v saline) 30 minutes prior to testing, in the same design as was used for the tolcapone stage.

6. Free-feeding stage (Days 26-29): At the end of the final day of the amphetamine manipulation, mice were placed back on free food and allowed to recover their weight for one day. Mice were then tested for three days under standard conditions, to assess performance under conditions of reduced motivation to perform for an appetitive reward.

It is possible to derive numerous measures from the 5CSRTT. However, to minimise problems of multiple testing, we have analysed only two: choice accuracy (defined as correct trials expressed as a percentage of total trials on which the mouse responded) and %correct (defined as correct trials expressed as a percentage of all trials [i.e. including omissions]). 5CSRTT data were analysed using repeated-measures analysis of variance (ANOVA). Performance across the first 6 days, comprising the short SD and Long ITI manipulations, was analysed within a single analysis. The counterbalancing precluded the use of this approach for later stages; therefore, these were analysed separately, and the impact of each manipulation (and its respective control) was expressed as a percentage of performance on the appropriate control day. For example, in the case of tolcapone, there were four within-subjects measures (performance on tolcapone and the following day, expressed as a percentage of performance on the day prior to tolcapone administration, and performance on vehicle and the following day, expressed as a percentage of performance on the day prior to vehicle administration). For these measures, drug (tolcapone or not) and day (the day of manipulation or the following day) were included as within-subjects factors. Stages 3-5 were analysed using this approach. For the free-feeding stage, only day was included as a within-subjects factor. Performance on days prior to each manipulation is presented in Supplementary Table 3.

**Supplementary Results**

The sections below provide statistical details of behavioural tests not discussed in the main text. All reliable findings involving genotype are reported in the main text, and details of sex effects (where these were observed) are reported below. Details on the cohorts used for behavioural testing are presented in Supplementary Table 4.

*No sex or genotype effects on locomotor activity*

COMT-Met mice showed no differences in locomotor activity to wild-types that could confound the results of subsequent behavioural tests (Supplementary Figure 1). Thus, whilst all mice showed habituation to a novel testing environment, indicated by a main effect of time (F_14.6,717.7_=101.1, p<0.001), there were no main or interactive effects of genotype, sex, time or cohort (F’s<2.9; p’s>0.1), other than a trend level cohort*time interaction (F_14.6,717.7_=1.6; p=0.061).

*Few sex differences and no genotype effects on performance of anxiety tests*

There was a main effect of sex on duration spent in the central region of the open field (F_1,56_=67.1; p<0.001). However, there were no other main or interactive effects in any of the tasks (F’s<1.8, p’s>0.2).

*Few sex or genotype effects on performance of learning and memory tests*

Other than a main effect of day on reference memory Y performance (F_3.7,139.2_=79.6, p<0.001), reflecting improvements in the mice’s performance of the task over time, and a day*genotype*sex interaction in the same task (F_3.7,139.2_=2.9, p=0.029; due to opposite effects of genotype in the two sexes on Day 2 [female wild type > female COMT-Met mice: p=0.024; male COMT-Met > male wild-type mice: p=0.094]), there were no other main or interactive effects on any of the novelty- or appetitively-motivated memory tasks (F’s<2.2, p’s>0.14).

COMT-Met mice showed no reliable changes in performance on the Morris water maze task. Mice learnt the location of the escape platform in both the acquisition and reversal stages, as reflected in main effects of day (acquisition: F_11,418_=76.4; p<0.001; reversal: F_2,76_=50.3; p<0.001) and trials within these days (acquisition: F_3,114_=9.7; p<0.001; reversal: F_3,114_=28.1; p<0.001) on the path length to the escape platform for both stages, as well as interactions between these factors (acquisition: F_33,1254_=3.2; p<0.001; reversal: F_6,228_=8.1; p<0.001). However, there were no main effects or interactions involving genotype for either acquisition or reversal (F’s<1.6; p’s>0.11) other than a day*trial*genotype*sex interaction for the path length taken to find the escape platform during acquisition (F_33,1254_=1.7; p=0.017). Similarly, there were no main or interactive effects of genotype for either training or reversal probe trials (spatial preference: F’s<1.6; p’s>0.22; accuracy of platform crossings : F’s<1.9; p’s>0.16; Supplementary Figure 4), other than a trend-level sex*day*genotype interaction (F_3,114_=2.7; p=0.076) for probe trial spatial preference, due to a sex difference in wild-type mice that was limited to the second probe trial.

There was a day*sex interaction (F_11,114_=3.7; p<0.001), and a trend level effect of sex (F_1,33_=3; p=0.076) on acquisition during the training phase of the Morris water maze. There were no other main effects or interactions for the acquisition of the training (F’s<1.9; p’s>0.18) or reversal (F’s<1.5; p’s>0.22) stages. Performance differed between Morris water maze training probe trials (main effect of day: spatial preference: F_3,114_=25.7; p<0.001; accuracy of platform crossings: F_2,76_=7.4; p=0.001), and there was a main effect of sex during the training phase probe trials (males outperformed females; spatial preference: F_1,38_=5.2; p=0.029; accuracy of platform crossings: F_1,38_=5.7; p=0.023), but there were no other main or interactive effects (F’s<1.4; p’s>2.5) on any measure of probe trial performance.

*5 Choice Serial Reaction Time Task*

COMT-Met and wild-type mice were well-matched in terms of free-feeding weight (Male wild-type: 28.7±0.8g [mean ± SEM]; Male COMT-Met: 28.6±0.8g; Female wild-type: 22.7±0.7g; Female COMT-Met: 22.2±0.7g; main effect of genotype: F_1,29_=0.14; p=0.709; sex*genotype: F_1,29_=0.06; p=0.806; main effect of sex: F_1,29_=64.8; p<0.001) and were maintained at 85% of this value for all stages of the 5CSRTT (except the final, free-feeding stage). Although male mice were heavier than females (main effect of sex: F_1,29_=64.8; p<0.001) there were no genotype differences (main effect of genotype: F_1,29_=0.14; p=0.709; sex*genotype: F_1,29_=0.06; p=0.806).

*Sex differences but no genotype differences were present during training*

Mice steadily improved their performance over training, reflected in a main effect of day (choice accuracy: F_41,1189_=43.9; p<0.001; %correct: F_41,1189_=29.0; p<0.001). There were main effects of sex for both measures (females outperforming males; choice accuracy: F_1,29_=11.5; p=0.002; %correct: F_1,29_=12.0; p=0.002) and a sex*day effect for the %correct measure (F_41,1189_=1.4; p=0.05), due to the presence of sex effects late in training (Days 32-42: p’s=0.001-0.075) that were largely absent during early training (Days 1-9:p’s=0.09-0.92). There were no other main or interactive effects (F’s<1.1; p’s>0.43).

*COMT-Met and wild-type mice show similar responses to injection stress in the 5CSRTT in the absence of sex differences*

A saline injection was administered to investigate the impact of mild, acute stress on 5CSRTT performance (Supplementary Figure 6); however, this was little affected by COMT genotype. The %correct measure was sensitive to the effect of injection stress, but effects of genotype were not robust. Thus, saline injection led to a decrease in %correct performance, reflected in a day (day of, vs day after, manipulation) by condition (injection or no injection) interaction (F_1,28_=9.0, p<0.001), due to a decrease in %correct performance on the injection day, compared to all other days (p’s<0.001). There were also main effects of day (F_1,28_=6.2; p=0.019) and condition (F_1,28_=6.9; p=0.014) but no other effects on this measure (F’s<2.4; p’s>0.13). There were no main or interactive effects on choice accuracy (F’s<1.6, p’s>0.22), other than a trend-level condition*genotype interaction (F_1,28_=3.0; p=0.092), due to a trend-level genotype difference after saline injection (COMT-Met mice maintained their performance to a greater extent than wild-types after injection; p=0.064), that was absent for the no-injection control (p=0.69).

*Sex differences during the tolcapone stage of the 5CSRTT*

There were subtle sex differences during the tolcapone stage of the task, but these were largely independent of genotype. There was a day*sex interaction (F_1,29_=7.5; p=0.010) for the %correct measure, due to a day effect in female mice (day of injection<post-injection day; p=0.006) that was absent in males (p=0.294). There was a trend-level day *sex*genotype interaction (F_1,29_=3.6; p=0.069) due to the day *sex interaction being most prominent in wild-type females (p=0.006) but the opposite day difference being present at trend-level in wild-type males (p=0.076) in the absence of day effects in COMT-Met mice of either sex (p’s>0.21). There were no other main effects or interactions (F’s<1.8; p’s>0.19).

*Amphetamine did not affect % correct or choice accuracy on the 5CSRTT in COMT-Met mice or wild-types*

Amphetamine (1.0mg/kg i.p.) did not have robust effects on either the %correct or choice accuracy measures over that of its vehicle. There were no main or interactive effects involving drug (i.e. amphetamine vs. vehicle) for choice accuracy (F’s<2.7; p’s>0.11) or %correct (F’s<2.0; p’s>0.17), other than a trend-level sex*drug interaction for choice accuracy (F_1,29_=3.1; p=0.087). However, this appeared to be due to a trend-level sex difference after administration of vehicle (p=0.069; female<male) that was absent after amphetamine (p=0.224). There was a day*genotype interaction for choice accuracy (F_1,29_=6.5; p=0.016; but not %correct: F_1,29_=1.0; p=0.33; Supplementary Figure 6) due to lower performance on the injection days compared to the days after injection in wild-type mice (p=0.001) that was absent in COMT-Met mice (p=0.907). However, there were no other main or interactive effects involving genotype (F’s< 1.8; p’s>0.19) for either measure.

During the amphetamine stage there was a main effect of sex on %correct (F_1,29_=6.3; p=0.018; males>females) but not choice accuracy: F_1,29_=0.09; p=0.77). There were main effects of day on both choice accuracy (F_1,29_=5.7; p=0.024) and %correct (F_1,29_=20.0; p<0.001) measures, due in both cases to poorer performance on the injection days than the post-injection days.

*Sex but no genotype effects on 5CSRTT performance in free-feeding mice*

Since a previous study demonstrated altered 5CSRTT performance following *ad libitum* exposure to food in the home cage prior to testing in COMT knockout mice compared with wild-types , we examined the performance of the COMT-Met mice under these conditions (Supplementary Figure 6). However, there were no main or interactive effects of genotype on either measure (F’s<1.3; p’s>0.29).

Consistent with the previous report , we found a main effect of sex on %accuracy (F_1,29_=5.3; p=0.029), present at trend level for the %correct measure (F_1,29_=3.5; p=0.071), due to females outperforming male mice under free-feeding conditions. For the choice accuracy measure, there was a main effect of day (F_1.9,55.9_=13.0; p<0.001), due to reduced accuracy on Day 3 compared to both other days (p’s<0.001), but no difference between Days 2 and 3 (p=0.701). There was also a trend day*sex interaction (F_1.9,55.9_=2.5; p=0.091) in addition to the main effect of sex described in the main text. There were no other main effects of interactions (F’s<1.3; p’s>0.29). For the %correct measure, there was a main effect of day (F_1.9;55.1_=29.5; p<0.001) – all days differed significantly from one-another (p’s<0.001) – as well as the trend effect of sex reported in the main text. There were no other main or interactive effects (F’s<1.8; p’s>0.17).

**Supplementary Tables**

Supplementary Table 1: Expression of genes in the 22q11DS critical deletion region is unchanged in COMT-Met vs. wild-type mice. Red shading indicates a nominal fold change greater than 1.0; blue shading indicates a nominal fold change of less than 1.0. Nominally significant changes in expression are indicated with asterisks.

|  | Frontal cortex | | Dorsal striatum | | Nucleus accumbens | |
| --- | --- | --- | --- | --- | --- | --- |
| Gene locus | Fold change | P (uncorr) | Fold change | P (uncorr) | Fold change | P (uncorr) |
| Dgcr2 | 1.0 | 0.673 | -1.1 | 0.495 | 1.0 | 0.549 |
| Tssk1 | +1.1 | 0.320 | +1.1 | 0.596 | -1.2 | 0.140 |
| Dgcr14 | -1.2 | 0.119 | 1.0 | 0.627 | +1.2 | 0.049* |
| Gsc2 | +1.1 | 0.534 | 1.0 | 0.712 | 1.0 | 0.894 |
| Slc25a1 | -1.2 | 0.204 | -1.1 | 0.555 | -1.1 | 0.261 |
| Vpreb2 | 1.0 | 0.577 | 1.0 | 0.684 | -1.1 | 0.482 |
| Dgcr6 | 1.0 | 0.807 | 1.0 | 0.666 | 1.0 | 0.872 |
| Prodh | -1.1 | 0.171 | -1.2 | 0.076 | +1.1 | 0.299 |
| Rtn4r | -1.2 | 0.114 | -1.2 | 0.034* | +1.2 | 0.349 |
| Zdhhc8 | 1.0 | 0.896 | -1.2 | 0.081 | 1.0 | 0.705 |
| Ranbp1 | 1.0 | 0.840 | 1.0 | 0.832 | 1.0 | 0.962 |
| Trmt2a | +1.4 | 0.011* | 1.0 | 0.982 | -1.1 | 0.327 |
| Dgcr8 | 1.0 | 0.564 | 1.0 | 0.798 | 1.0 | 0.703 |
| Tango2 | 1.0 | 0.851 | -1.1 | 0.106 | +1.1 | 0.273 |
| Arvcf | -1.1 | 0.402 | 1.0 | 0.591 | 1.0 | 0.663 |
| **Comt** | **-1.6** | **0.001*** | **-1.4** | **0.009*** | **-1.4** | **0.001*** |
| Txnrd2 | -1.1 | 0.338 | 1.0 | 0.794 | -1.2 | 0.227 |
| Gnb1l | 1.0 | 0.830 | 1.0 | 0.668 | +1.2 | 0.053 |
| Tbx1 | 1.0 | 0.944 | -1.1 | 0.243 | 1.0 | 0.556 |
| Gp1bb/ Sept5 | -1.1 | 0.196 | 1.0 | 0.956 | -1.1 | 0.384 |
| Cldn5 | -1.1 | 0.516 | +1.1 | 0.552 | +1.1 | 0.387 |
| Cdc45 | +1.2 | 0.216 | 1.0 | 0.843 | -1.1 | 0.330 |
| Ufd1l | 1.0 | 0.725 | 1.0 | 0.918 | 1.0 | 0.612 |
| Mrpl40 | -1.1 | 0.495 | -1.1 | 0.269 | +1.1 | 0.427 |
| Hira | +1.1 | 0.481 | 1.0 | 0.912 | 1.0 | 0.766 |

Supplementary table 2: Tissue levels of dopamine, 5-hydroxytryptamine and 5- hydroxyindoleacetic acid in wild-type and COMT-Met mice. Data are presented as means ± SEMs and were compared using Mann Whitney-U tests.

| Region | Dopamine | | | 5-hydroxytryptamine | | | 5- hydroxyindoleacetic acid | | |
| --- | --- | --- | --- | --- | --- | --- | --- | --- | --- |
|  | Wild-type | COMT-Met | *p* | Wild-type | COMT-Met | *p* | Wild-type | COMT-Met | *p* |
| Medial prefrontal cortex | 38.3 ± 3.4 | 42.4 ± 3.9 | *0.52* | 76.1 ± 14.5 | 72.8 ± 7.7 | *0.99* | 28.7 ± 5.4 | 40.8 ± 5.0 | *0.18* |
| Nucleus accumbens | 894.5 ± 173.1 | 717.2 ± 98.9 | *0.73* | 79.3 ± 15.7 | 69.6 ± 12.2 | *0.62* | 26.0 ± 5.0 | 28.8 ± 5.25 | *0.99* |
| Dorsal striatum | 1212.2 ± 103.4 | 1403.2 ± 139.3 | *0.47* | 31.0 ± 3.4 | 24.7 ± 3.7 | *0.18* | 8.6 ± 2.0 | 12.1 ± 2.0 | *0.21* |
| Dorsal hippocampus | 5.4 ± 1.1 | 7.0 ± 1.2 | *0.43* | 48.8 ± 6.4 | 47.4 ± 5.1 | *0.99* | 32.9 ± 3.3 | 31.5 ± 3.1 | *0.91* |
| Ventral hippocampus | 10.1 ± 1.3 | 44.1 ± 28.2 | *0.39* | 108.7 ± 12.4 | 151.8 ± 37.5 | *0.90* | 60.9 ± 6.8 | 112.5 ± 36.0 | *0.99* |

Supplementary Table 3: 5CSRTT baseline and pre-manipulation performance data. Data are presented as means ± SEMs and were analysed by ANOVAs, as detailed in the Supplementary Methods.

| Day | Choice accuracy | | | %Correct | | |
| --- | --- | --- | --- | --- | --- | --- |
|  | Wild-type | COMT-Met | Main effect of genotype | Wild-type | COMT-Met | Main effect of genotype |
| End of training | 90.7 ± 1.22 | 92.2 ± 1.25 | F1,29=0.7; p=0.41 | 71.6 ± 2.12 | 72.5 ± 2.17 | F_1,29_=0.1; p=0.76 |
| Pre-short SD | 93.1 ± 1.29 | 91.4 ± 1.32 | F1,29=0.9; p=0.36 | 75.5 ± 2.93 | 70.1 ± 3.00 | F_1,29_=1.7; p=0.21 |
| Pre-long ITI | 90.8 ± 1.16 | 92.5 ± 1.18 | F1,29=1.7; p=0.31 | 75.8 ± 2.13 | 74.8 ± 2.18 | F_1,29_=0.1; p=0.76 |
| Pre-Injection stress | 94.8 ± 1.03 | 93.9 ± 1.10 | F,128=0.4l p=0.56 | 75.4 ± 2.40 | 75.1 ± 2.57 | F_1,28_=0.005; p=0.94 |
| Pre-Injection stress control | 93.9 ± 0.70 | 95.5 ± 0.74 | F1,28=2.4; p=0.14 | 75.0 ± 2.30 | 78.9 ± 2.46 | F_1,28_=1.3; p=0.27 |
| Pre-tolcapone | 92.8 ± 0.97 | 94.2 ± 1.00 | F1,29=1.1; p=0.30 | 74.5 ± 2.17 | 79.2 ± 2.22 | F_1,28_=2.3; p=0.14 |
| Pre-tolcapone vehicle | 93.0 ± 1.02 | 94.0 ± 1.05 | F1,29=0.5; p=0.49 | 77.6 ± 2.49 | 76.8 ± 2.55 | F_1,29_=0.1; p=0.80 |
| Pre-amphetamine | 93.1 ± 1.11 | 95.7 ± 1.13 | F1,29=2.8; p=0.10 | 79.2 ± 2.09 | 81.4 ± 2.14 | F_1,29_=0.5; p=0.48 |
| Pre-amphetamine vehicle | 93.7 ± 0.76 | 95.8 ± 0.78 | F1,29=3.7; p=0.06 | 80.8 ± 2.01 | 80.1 ± 2.06 | F_1,29_=0.1; p=0.81 |
| Pre-free feeding | 94.1 ± 0.87 | 96.5 ± 0.89 | F1,29=3.8; p=0.06 | 79.3 ± 2.55 | 83.4 ± 2.61 | F_1,29_=1.3; p=0.27 |

**Supplementary Table 4: cohorts used for behavioural testing**

| **Cohort** | **Number of backcrosses since Prm-Cre cross** | **Number of wild-type mice (female/male)** | **Number of COMT-Met mice (female/male)** | **Behavioural tasks completed, in order** |
| --- | --- | --- | --- | --- |
| **1** | **5** | 10 (10M/0F) | 10 (10M/0F) | Elevated plus maze  Light-dark box  Open field  Novelty suppressed feeding  Spatial novelty preference  Photobeam activity monitoring |
| **2** | **8** | 17 (10F/7M) | 22 (13F/9M) | Spontaneous alternation  Spatial novelty Y-maze  Photobeam activity monitoring  Novel object recognition  Morris water maze |
| **3** | **8** | 20 (12 F/8M) | 22 (10F/12M) | Elevated plus maze  Open field  Light/dark box  Novelty suppressed feeding  Reference memory Y maze |
| **4** | **10** | 20 (10F/10M) | 20 (10F/10M) | Five choice serial reaction time task |

**Supplementary figure legends**

Supplementary Figure 1: Correlation plot of microarray results. The plot shows a heatmap of correlations between individual samples from wild-type (WT) and COMT-Met (Met) within the microarray experiment. Samples from the same region show high correlation coefficients. Samples from the dorsal striatum (dStr) and nucleus accumbens (NAc) show moderately strong correlations, whilst correlations between these regions and medial prefrontal cortex (mPFC) are weak. Red colouring indicates samples with similar expression levels whereas black indicates samples with significantly different expression levels.

Supplementary Figure 2: COMT-Met mice and wild-types perform similarly on tests of anxiety-related behaviours. COMT-Met mice (red) and wild-type mice (blue) spend similar amounts of time (a) in the open arms of the elevated plus and (b) in the light compartment of light-dark box. COMT-Met and wild-type mice show similar (c) latencies to enter and, (d) time in, the central region of the open field. (e) COMT-Met and wild-type mice show a similar duration between initial contact with and consumption of a novel food. (n=30 wild-type [18 male]; n=32 COMT-Met [22 male]).

Supplementary Figure 3: COMT-Met mice perform similarly to wild-type mice on cognitive tasks. COMT-Met mice (red) and their wild-type littermates (blue) performed similarly on (a) the novel object recognition and (b) spontaneous alternation tasks (for which chance performance is shown as a dotted line in each case and n=30 wild-type [18 male]; n=32 COMT-Met [22 male] mice), and (c) the reference memory Y maze (n=17 wild-type [7 male]; n=22 Met mice [ 9 male]). For (c) the asterisk indicates p<0.05 day*genotype*sex interaction, detailed further in the Supplementary Results section.

Supplementary Figure 4: COMT-Met and wild-type mice show similar performance on the Morris water maze. There was little evidence for genotype differences between COMT-Met (red) and wild-type (blue) mice (n=17 wild-type [7 male]; n=22 Met mice [9 male]) on the path length to find the platform during (a) initial training or (b) reversal stages. Neither were there differences in (c) spatial preference or (d) accuracy of platform crossings during the probe trials in which the platform was removed. The asterisks in (c) indicate p<0.001 for the post hoc test of the sex difference in wild-type mice only driving the sex*day*genotype interaction for spatial preference described in the main text.

Supplementary Figure 5: COMT-Met and wild-type mice performed similarly during training on the 5CSRTT. There were no differences between the performance of COMT-Met (red; n=20 [10F/10M]) and wild-type (blue; n=20 [10F/10M]) mice during the training stages of the 5CSRTT for either (a) choice accuracy or (b) %correct measures.

Supplementary Figure 6: Genotype had little impact on performance of the injection stress, amphetamine or free-feeding stages of the 5CSRTT. COMT-Met (Met) and wild-type (WT) mice responded similarly to a saline injection (Sal) challenge, compared to a no injection (No Inj) control day for both (a) choice accuracy and (b) %correct measures. (c) On the amphetamine stage, there was a day*genotype interaction for the choice accuracy measure due to differences between performance on the injection day (green) vs. the day after injection (purple) in wild-type mice (p=0.001, indicated with asterisks) that was not seen in COMT-Met mice. However, this did not interact with drug (amphetamine [AMPH] vs. vehicle [Veh]) and so seemed to be non-specific response to injection. (d) There were no genotype differences, nor interactions, for the %correct measure on the amphetamine stage. There were no differences in the performance of COMT-Met (red) and wild-type (blue mice) for either (e) choice accuracy or (f) %correct following a return to free feeding, although male (lighter colours) and female (darker colours) responded differently to this manipulation, as detailed in the Supplementary Results. ***p<0.001.

**Supplementary references**

Bannerman DM, Niewoehner B, Lyon L, Romberg C, Schmitt WB, Taylor A*, et al* (2008). NMDA receptor subunit NR2A is required for rapidly acquired spatial working memory but not incremental spatial reference memory. *J Neurosci* **28**(14): 3623-3630.

Borges N, Vieira-Coelho MA, Parada A, Soares-da-Silva P (1997). Studies on the tight-binding nature of tolcapone inhibition of soluble and membrane-bound rat brain catechol-O-methyltransferase. *J Pharmacol Exp Ther* **282**(2): 812-817.

Lane TA, Boerner T, Bannerman DM, Kew JN, Tunbridge EM, Sharp T*, et al* (2013). Decreased striatal dopamine in group II metabotropic glutamate receptor (mGlu2/mGlu3) double knockout mice. *BMC Neurosci* **14**(1): 102.

Papaleo F, Crawley JN, Song J, Lipska BK, Pickel J, Weinberger DR*, et al* (2008). Genetic dissection of the role of catechol-O-methyltransferase in cognition and stress reactivity in mice. *J Neurosci* **28**(35): 8709-8723.

Papaleo F, Erickson L, Liu G, Chen J, Weinberger DR (2012). Effects of sex and COMT genotype on environmentally modulated cognitive control in mice. *Proc Natl Acad Sci U S A* **109**(49): 20160-20165.

Tammimaki A, Aonurm-Helm A, Kaenmaki M, Mannisto PT (2016). Elimination of extracellular dopamine in the medial prefrontal cortex of conscious mice analysed using selective enzyme and uptake inhibitors. *J Physiol Pharmacol* **67**(2): 301-309.

Tunbridge EM, Weickert CS, Kleinman JE, Herman MM, Chen J, Kolachana BS*, et al* (2007). Catechol-o-Methyltransferase Enzyme Activity and Protein Expression in Human Prefrontal Cortex across the Postnatal Lifespan. *Cereb Cortex*.
