# Supplementary figures and images for: Genotype-Dependent Effects of COMT Inhibition on Cognitive Function in a Highly Specific, Novel Mouse Model of Altered COMT Activity
Source: Neuropsychopharmacology. 2016 Aug 10;41(13):3060–9. doi: 10.1038/npp.2016.119 (PMC5101554; doi:10.1038/npp.2016.119)

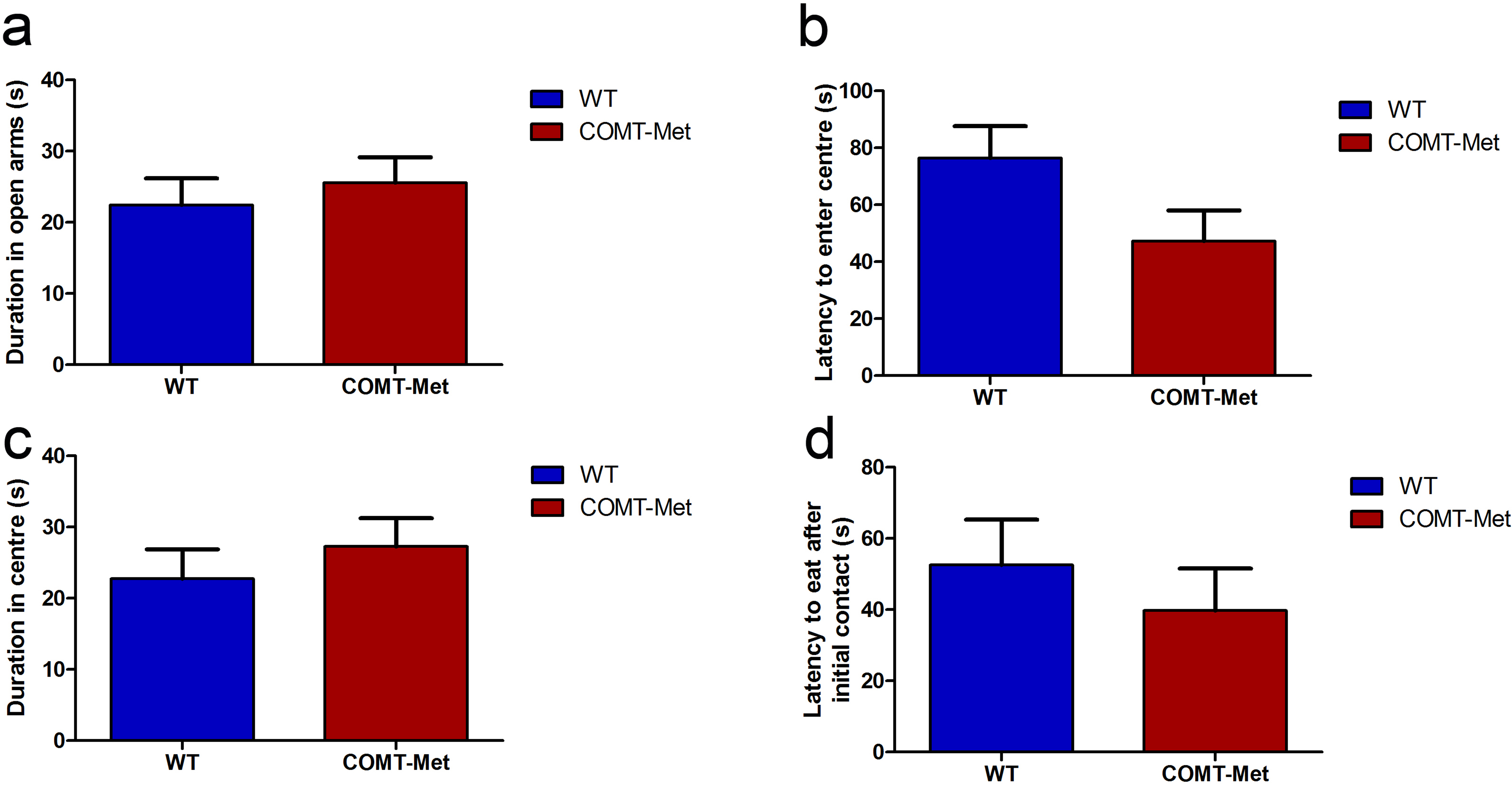

Supplement: Supplementary Figure 2 [file npp2016119x3.tif]

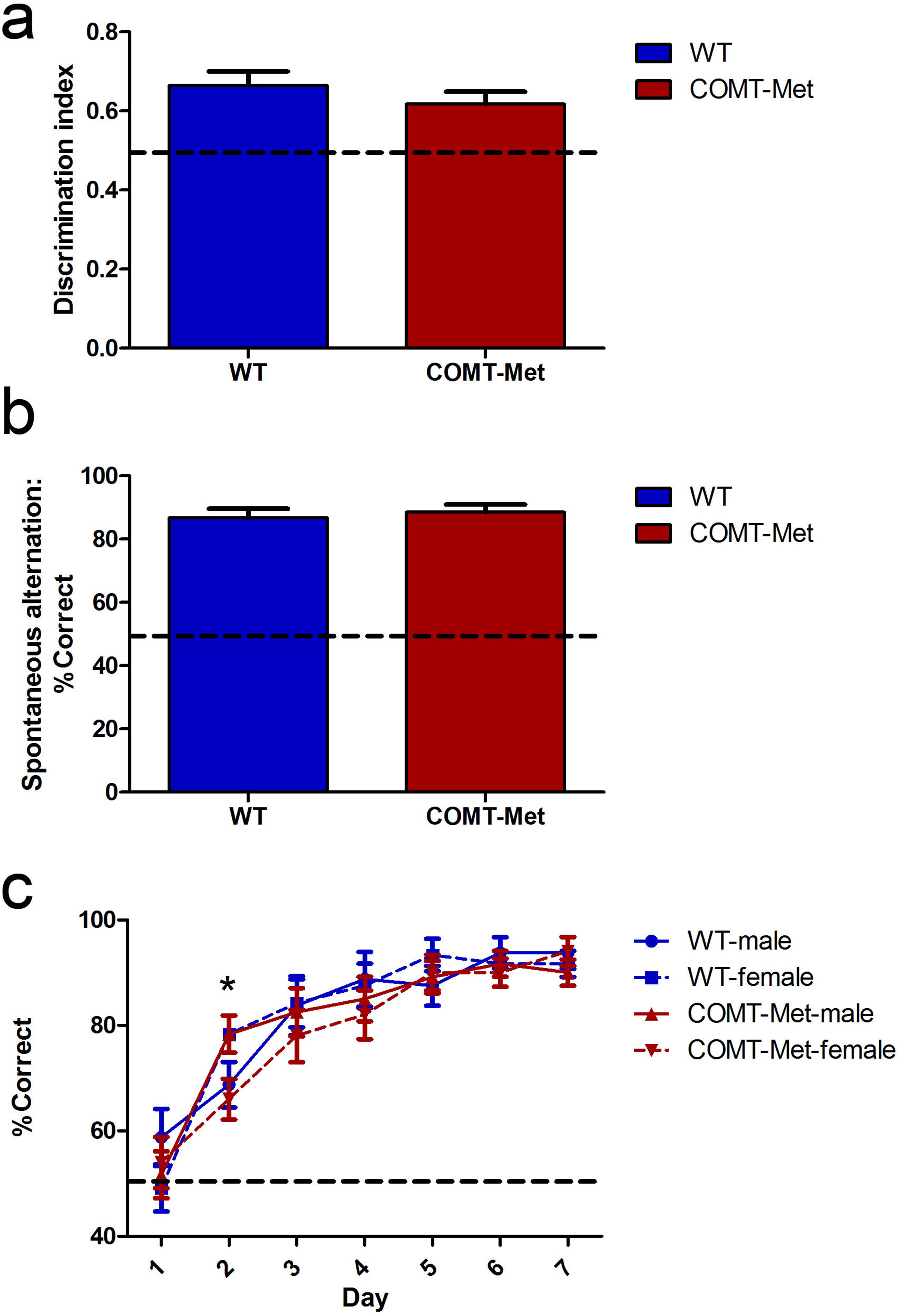

Supplement: Supplementary Figure 3 [file npp2016119x4.tif]

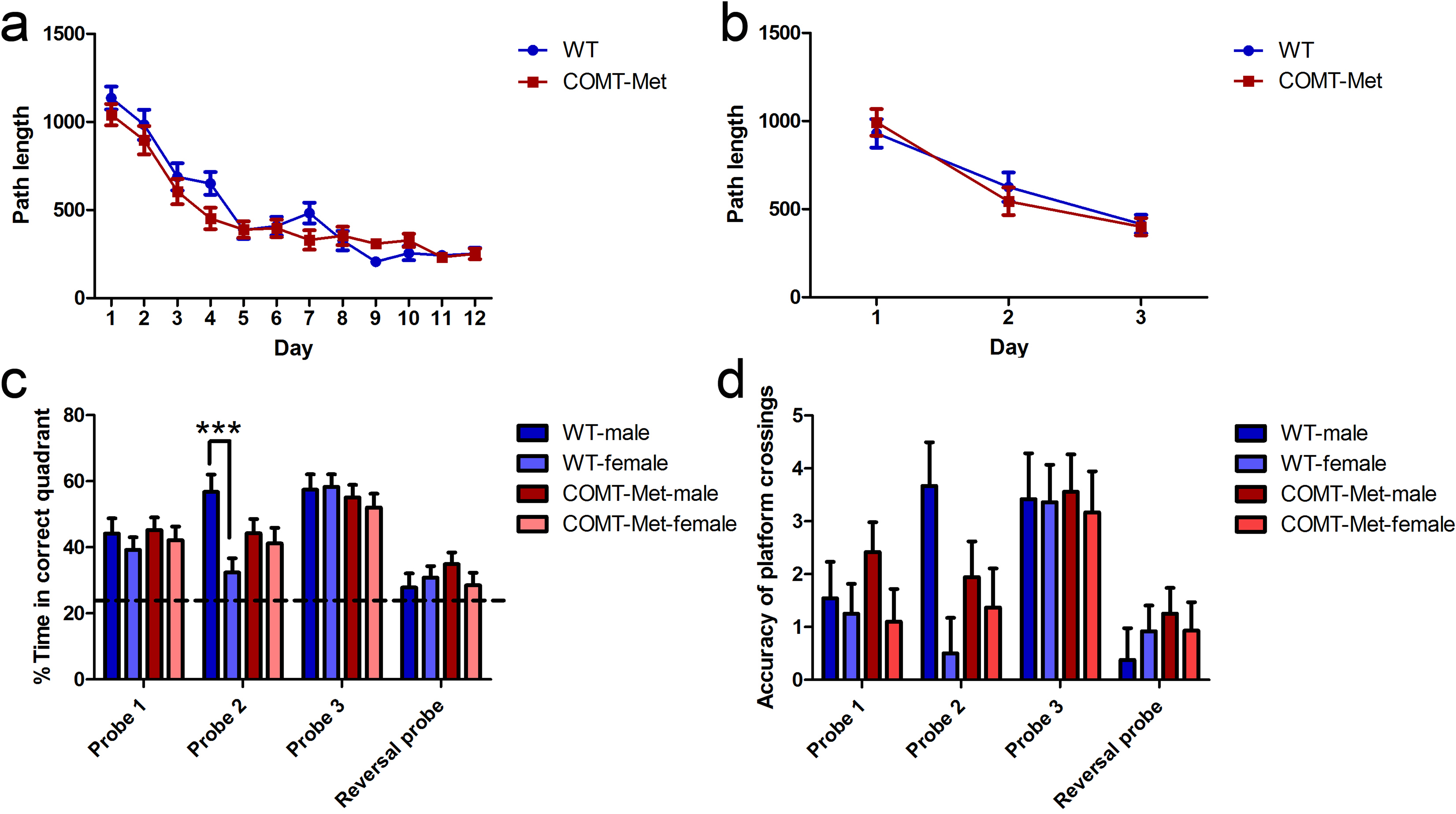

Supplement: Supplementary Figure 4 [file npp2016119x5.tif]

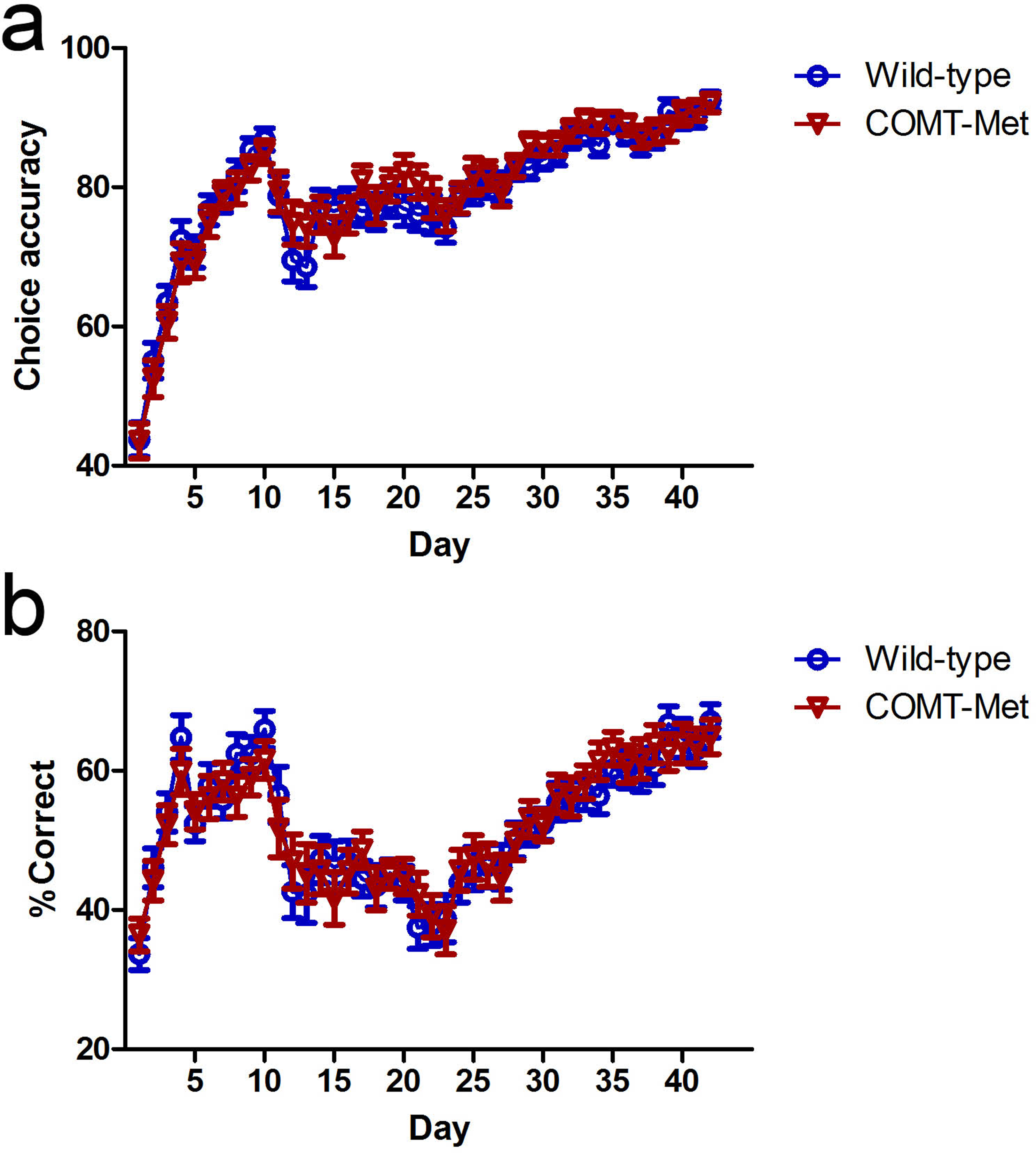

Supplement: Supplementary Figure 5 [file npp2016119x6.tif]

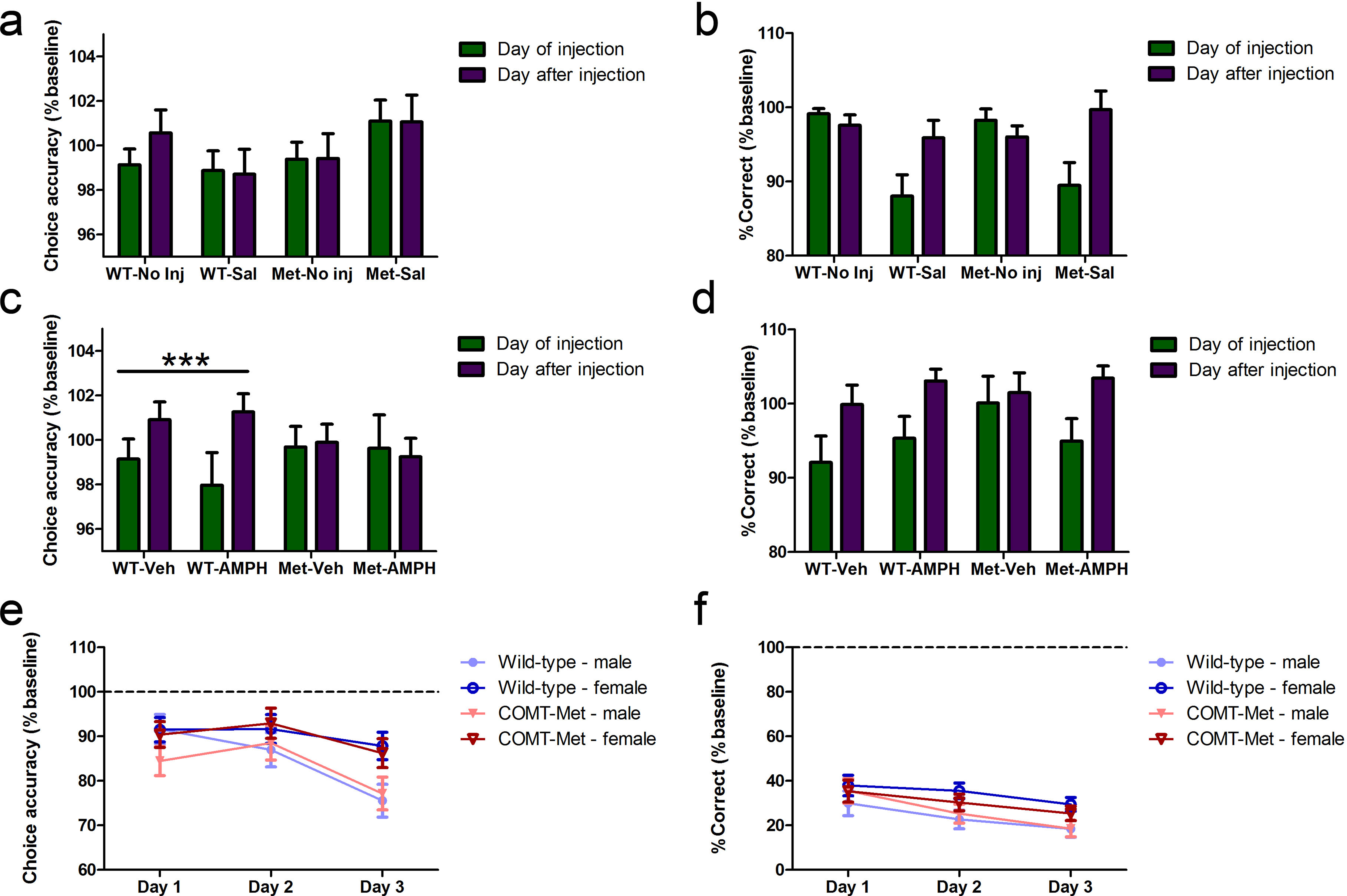

Supplement: Supplementary Figure 6 [file npp2016119x7.tif]
